# Importance of absorbable surgical sutures for the prevention of stitch abscess after surgery in patients with oral squamous cell carcinoma

**DOI:** 10.4317/medoral.21445

**Published:** 2017-04-08

**Authors:** Noriaki Yamamoto, Yoshihiro Takahashi, Tatsuyuki Kono, Ayaka Abe, Kazuhiro Kawamura, Takaaki Joujima, Nao Wakasugi-Sato, Shun Nishimura, Masafumi Oda, Tatsurou Tanaka, Shinji Kito, Kenji Kawano, Yasuhiro Morimoto

**Affiliations:** 1 DDS PhD, Department of Dentistry and Oral-Maxillo-Facial Surgery, Oita University, Oita, Japan; 2 DDS, Department of Dentistry and Oral-Maxillo-Facial Surgery, Oita University, Oita, Japan; 3 DDS, Division of Oral and Maxillofacial Radiology, Kyushu Dental University, Kitakyushu, Japan; 4 DDS PhD, Division of Oral and Maxillofacial Radiology, Kyushu Dental University, Kitakyushu, Japan; 5 DDS PhD, Center for Oral Biological Research, Kyushu Dental University, Kitakyushu, Japan

## Abstract

**Background:**

To elucidate the significance of absorbable surgical sutures in the occurrence of stitch abscess after surgery in patients with oral squamous cell carcinoma (SCC).

**Material and Methods:**

The subjects were 251 patients who underwent excision and/or reconstruction and/or neck dissection for oral SCC using absorbable surgical sutures. Detection rates and characteristics of patients with stitch abscess were retrospectively evaluated by comparing between our present and previous data.

**Results:**

There was only one stitch abscess among the 251 patients. A significant difference in the incidence of stitch abscess was found between the present data and our previous data. Of course, no significant correlations were found between the occurrence of stitch abscess using absorbable surgical sutures and the various factors seen in our previous analysis.

**Conclusions:**

A complete switch of surgical sutures from silk to absorbable surgical sutures is needed for surgery in patients with oral SCC.

** Key words:**Stitch abscess, oral cancer, occurrence, absorbable surgical sutures, silk suture.

## Introduction

Stitch abscesses, which are abscesses that occur due to suture infections, are noteworthy complications after surgical procedures (1-5). After surgery for malignant tumors, it has been very difficult to differentiate stitch abscess from metastatic lymph nodes or local recurrence of primary tumor. In our previous study, we elucidated the usefulness of ultrasonography (US) for the exact diagnosis of stitch abscess, similar to the results of Hsu *et al.* (4-6); US could provide a precise diagnosis of stitch abscess (4-6). In addition, we demonstrated that the risk of stitch abscess in patients with oral cancers was related to age, liver dysfunction, and/or the presence of allergies (5).

Surgery with silk sutures increases the risk of infections because they react with the connective tissue, causing adhesions around the stitch (7). Certainly, based on the previous reports on the comparison between polyglycolic acid and silk, the use of silk sutures in surgical procedures is decreasing to prevent stitch abscesses in accidental wounds, rupture of the Achilles tendon, abdominal incisions, and hepatectomy (1-3,7,8). To the best of our knowledge, however, there have been no reports related to surgery in patients with oral squamous cell carcinoma (SCC).

In the present study, we examined whether the use of absorbable sutures in surgery for patients with oral SCC reduces the risk of stitch abscess. Detection rates and characteristics of stitch abscess in patients who underwent excision and/or reconstruction and/or neck dissection for oral SCC using absorbable surgical sutures for high ligation of the blood vessels were investigated. The present data were compared with our previous data (5).

## Material and Methods

The subjects were 251 patients (148 men, 103 women) who underwent excision and/or reconstruction and/or neck dissection for SCC of the oral cavity from 2011 to 2013 at the Department of Oral and Maxillofacial Surgery of Oita University Hospital. In all cases where the original operative information was available, absorbable surgical sutures, not silk sutures, were used for high ligation of the blood vessels. In this study, the Human Investigations Committee of Oita University Hospital protected individuals’ rights. Approval of the present study was obtained from the institutional review board of Oita University Hospital (No. 972).

All 251 patients were retrospectively divided into two groups based on the presence or absence of stitch abscess on images using various modalities mentioned below. Patients were examined by US at 1-month intervals after surgery for 1 year using a GE LOGIQ-e ultrasound machine (GE Healthcare, Milwaukee, WI). Computed tomography (CT) was performed at 3 months, 6 months, and 1 year after surgery with an Aquilion One (Toshiba Co. Ltd., Tokyo, Japan). Magnetic resonance imaging (MRI) was performed at 3 months, 6 months, and 1 year after surgery using a 1.5-T MAGNETOM Verio (Siemens AG, Erlangen, Germany). Additional US, CT, and MRI examinations were done as soon as possible if abnormal findings were detected on any regular examination. Positron emission tomography (PET)-CT using 18fluoro-2-deoxy-D-glucose (18F-FDG) was performed using a Biograph mCT40 (Siemens AG) if abnormal findings were detected on any examination. Characteristic findings on various imaging modalities such as US, CT, MRI, and PET-CT using 18F-FDG were also used as identified by Yamamoto *et al.* (4,5). Specifically, a hypoechoic mass was identified as the characteristic finding of stitch abscess on US. A soft tissue mass with/without central nodal necrosis was identified as the characteristic finding of stitch abscess on CT and MRI. Moreover, an 18F-FDG positive mass was identified as the characteristic finding of stitch abscess on PET-CT. The changes in stitch abscesses on subsequent US examinations were analyzed retrospectively. However, cases with masses and swelling that disappeared within 1 month and masses and swelling diagnosed as non-tumor recurrence and/or non-metastatic lymph nodes were excluded as non-stitch abscesses.

The patient groups with and without stitch abscesses were compared with respect to various factors to identify those that predispose to the occurrence of stitch abscess by the Chi-squared test. The factors analyzed included patients’ sex and age, chemother-apy treatment, radiotherapy treatment, the presence of allergy, and blood test results. In addition, the present data were compared with the previous data using the Chi-squared test (5).

All statistical analyses were performed using SPSS™ software, version 11 (SPSS Inc., Chicago, IL, USA). Results were considered significant at *p*<0.05.

## Results

- The incidence and imaging characteristics of stitch abscesses after surgery using absorbable surgical sutures in patients with oral SCC

**Figure 1:**
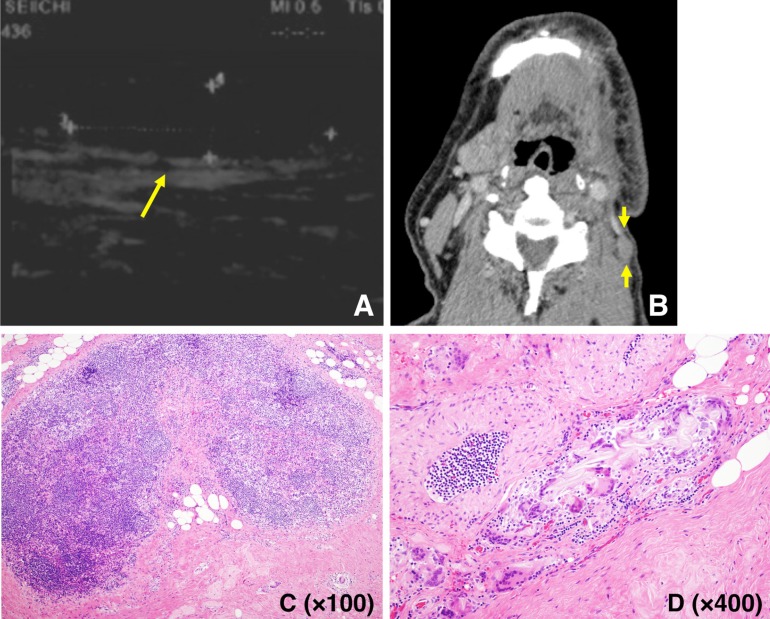
US (A), CT (B), and pathological specimen (C, D) of an 80-year-old man with a stitch abscess in the left submandibular space at 3 months after surgery for left upper gingiva carcinoma. The image demonstrates a hypoechoic mass on US (arrow) and a soft tissue mass on CT (arrow). On examination of the pathological specimen, surgical sutures and inflammatory findings are seen.

The primary site, age, and sex of patients with oral SCC are shown in  Table 1. The most common site was the tongue [119], with the upper gingiva [45] next. The age of patients with oral SCC ranged from 17 to 93 (mean: 68.3) years, with 148 male and 103 female patients. The overall 5-year survival rate was 66.8%. In addition, the occurrence rate of metastatic lymph nodes and the recurrence rate of primary tumors within 1 year were 17.5% and 13.1%, respectively. Overall, only one (0.4%) of the 251 patients was diagnosed as having a stitch abscess based on pathological findings ( Table 2). A hypoechoic mass was seen as a characteristic finding of stitch abscess on US, and a soft tissue mass was seen on CT (Fig. 1). The pathological specimen was diagnosed as a stitch abscess (Fig. 1). Surgical removal of the stitch abscess was performed, after which recurrence of the stitch abscess was not detectable.

**Table 1:**
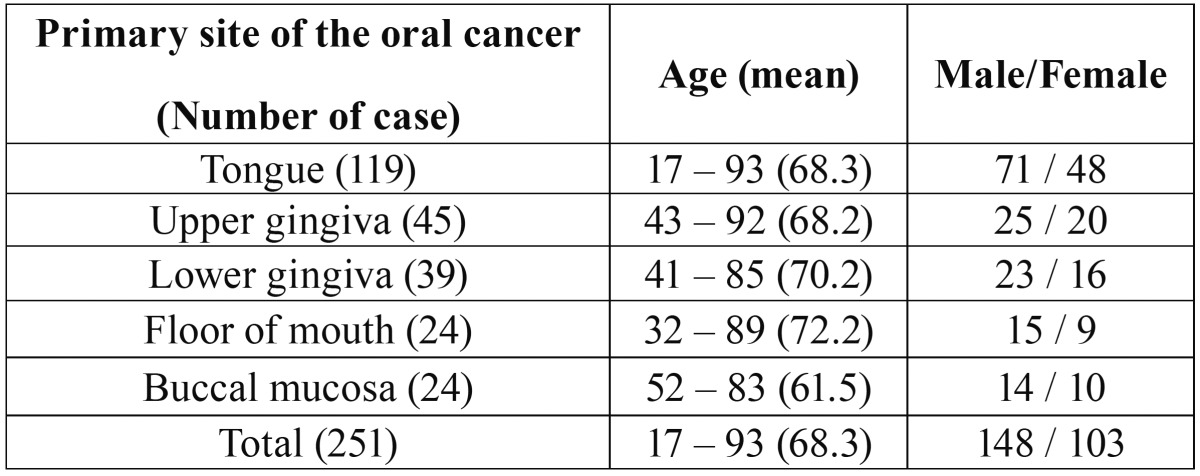
Patient characteristics.

**Table 2:**
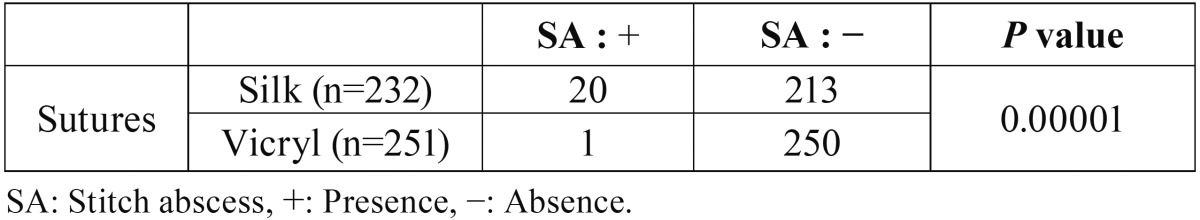
Difference in the incidence of stitch abscesses between silk surgical sutures and absorbable surgical sutures for high ligation of the blood vessels.

-Comparison of the incidence and factors predisposing to and characteristics of stitch abscesses after surgery in patients with oral SCC between the present data (using absorbable surgical sutures for high ligation of the blood vessels) and our previous data (5) (using silk surgical suture)

Unlike our previous data (5), the detection rate of stitch abscess was only 0.4% (1/251) using absorbable surgical sutures. In addition, a significant difference in the incidence of stitch abscess was found between the present data and our previous data ( Table 2) (χ2 test t; *p*=0.00001) (5).

The relationships between various factors and the occurrence of stitch abscesses using absorbable surgical sutures are shown in  Table 3. There were no significant differences in sex (χ2 test; *p*=0.403), the presence or absence of chemotherapy for oral cancer (χ2 test; *p*=0.438), and the presence or absence of radiotherapy for oral cancer (χ2 test; *p*=0.471), as in our previous data (5). However, no significant correlation was found between the occurrence of stitch abscess and over or under 60 years of age (χ2 test; *p*=0.597), a history of allergy (χ2 test; *p*=0.639), or liver dysfunction (χ2 test; *p*=0.639), unlike our previous data (5).

**Table 3:**
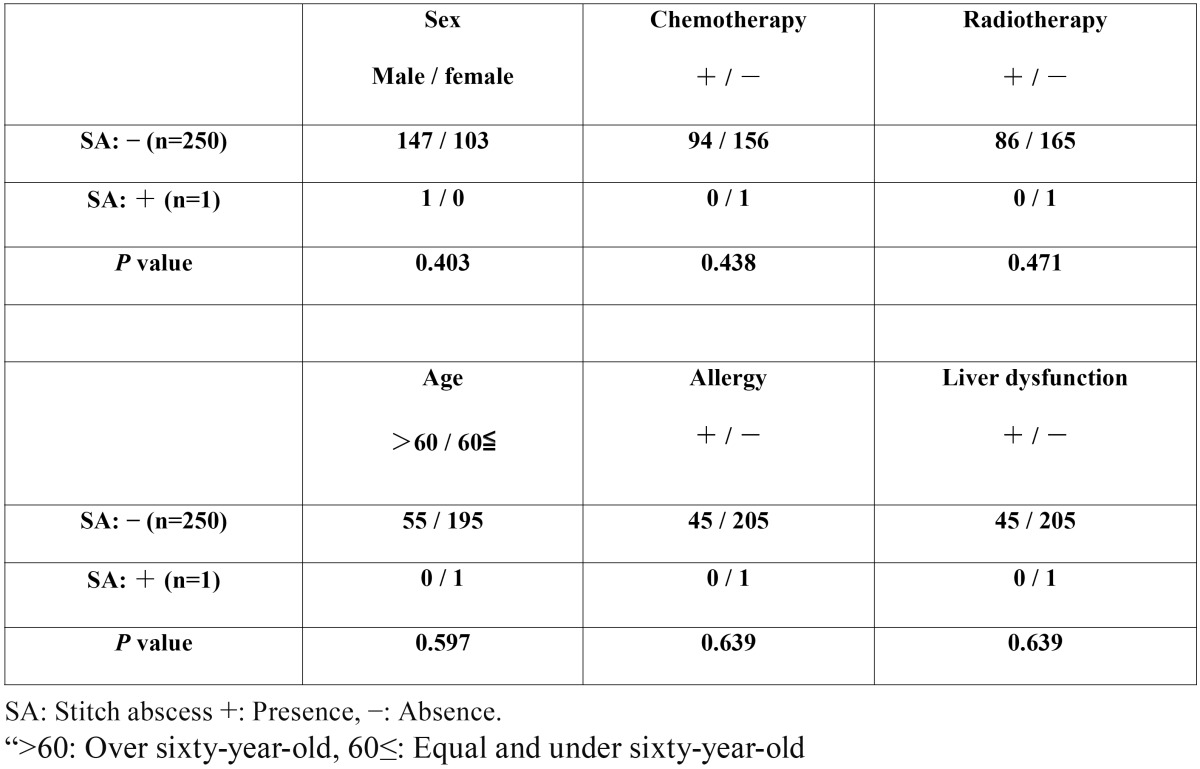
Relationships between various factors and the occurrence of stitch abscesses using absorbable surgical sutures.

## Discussion

The most interesting result of the present study was that a significant difference in the incidence of stitch abscess was found between the present data using absorbable surgical sutures for high ligation of the blood vessels in patients with oral SCC and our previous data using silk sutures (χ2 test; *p*=0.00001) (5). The present data suggest the commonly accepted theory that the use of silk sutures in surgical procedures is decreasing to prevent stitch abscesses for patients with oral SCC (1,2,7,8). In human gingival tissues, the degree of the inflammatory reaction varies with the suture material used (9). Silk suture also causes a more extensive inflammatory reaction than absorbable suture because of bacterial adherence (9-11). Therefore, using silk sutures should increase the occurrence of stitch abscesses in the oral mucosa. Based on the present evidence and the previous reports, we propose that oral and maxillofacial surgeons worldwide should immediately stop using silk sutures for high ligation of blood vessels in patients with oral SCC to prevent complications such as stitch abscesses (1,2,7-11). At the same time, the present information about the complications of silk sutures needs to be widely disseminated through reports on the occurrence of stitch abscess after oral cancer surgery, including dental surgery (9-11).

In addition, the other important result in the present study was that the stitch abscess based on pathological findings occurred in only one (0.4%) of the 251 oral SCC patients when absorbable surgical sutures were used for high ligation of the blood vessels. The reason for this might be that infection of the absorbable surgical suture may have occurred before it was absorbed. Certainly, there have been some reports of such cases (1-3,12,13). To the best of our knowledge, however, this is the first report of the use of absorbable surgical sutures being used for high ligation of the blood vessels after surgery for patients with oral SCC, though this may be done in other parts of the world. In the present case, the characteristic finding of stitch abscesses after surgery in patients with oral SCC, the presence of multiple stitch abscesses, could not be detected, unlike our previous reports (4,5). The possible explanation for this is that the use of absorbable surgical sutures made it difficult for stitch abscesses to occur after surgery in patients with oral SCC. The patient in the present case was not relatively young, and did not have a history of allergy and/or liver dysfunction reported as predisposition as in our previous studies (4,5). In addition, because a stitch abscess occurred in only one case, it was not possible to analyze the factors predisposing to the occurrence of stitch abscesses when absorbable surgical sutures were used for high ligation of the blood vessels in patients with oral SCC. We should now pay attention to stitch abscesses when absorbable surgical sutures are used for high ligation of the blood vessels in patients with oral SCC, and we should elucidate the factors predisposing to and the characteristics of stitch abscesses. Further study is needed to address this issue.

The present study had several limitations. First, the present study was different from the previous study in that the operators and the hospital were different (5). However, the first author was the same and is convinced that there were no important differences between the operators and the hospitals. For example, the overall 5-year survival rate was 82%, with a rate of 95% for T1N0M0 in the hospital of the previous report (5). In the present hospital, the overall 5-year survival rate was 85%, with a rate of 96% for T1N0M0. In addition, the occurrence rate of metastatic lymph nodes and the recurrence rate of primary tumors within 1 year also were 3% and 1%, respectively, in the previous hospital. Next, the sample size was relatively small, and only one patient developed a stitch abscess. Therefore, it was not possible to analyze the predisposing factors and characteristics of stitch abscesses.
